# Development and Clinical Translation of Approved Gene Therapy Products for Genetic Disorders

**DOI:** 10.3389/fgene.2019.00868

**Published:** 2019-09-25

**Authors:** Alireza Shahryari, Marie Saghaeian Jazi, Saeed Mohammadi, Hadi Razavi Nikoo, Zahra Nazari, Elaheh Sadat Hosseini, Ingo Burtscher, Seyed Javad Mowla, Heiko Lickert

**Affiliations:** ^1^Institute of Diabetes and Regeneration Research, Helmholtz Zentrum München, Neuherberg, Germany; ^2^Institute of Stem Cell Research, Helmholtz Zentrum München, Neuherberg, Germany; ^3^Stem Cell Research Center, Golestan University of Medical Sciences, Gorgan, Iran; ^4^Department of Molecular Genetics, Faculty of Biological Sciences, Tarbiat Modares University, Tehran, Iran; ^5^Metabolic Disorders Research Center, Golestan University of Medical Sciences, Gorgan, Iran; ^6^Infectious Disease Research Center, Golestan University of Medical Sciences, Gorgan, Iran; ^7^Department of Biology, School of Basic Sciences, Golestan University, Gorgan, Iran; ^8^Department of Nanobiotechnology, Faculty of Biological Sciences, Tarbiat Modares University, Tehran, Iran

**Keywords:** gene therapy, cell-based gene therapy, drug, genetic disease, clinic

## Abstract

The field of gene therapy is striving more than ever to define a path to the clinic and the market. Twenty gene therapy products have already been approved and over two thousand human gene therapy clinical trials have been reported worldwide. These advances raise great hope to treat devastating rare and inherited diseases as well as incurable illnesses. Understanding of the precise pathomechanisms of diseases as well as the development of efficient and specific gene targeting and delivery tools are revolutionizing the global market. Currently, human cancers and monogenic disorders are indications number one. The elevated prevalence of genetic disorders and cancers, clear gene manipulation guidelines and increasing financial support for gene therapy in clinical trials are major trends. Gene therapy is presently starting to become commercially profitable as a number of gene and cell-based gene therapy products have *entered the market and the clinic*. This article reviews the history and development of twenty approved human gene and cell-based gene therapy products that have been approved up-to-now in clinic and markets of mainly North America, Europe and Asia.

## Introduction

In medicine, gene therapy is defined as therapeutic strategy that transfers DNA to a patient’s cells to correct a defective gene or a gene product in order to treat diseases that are not curable with conventional drugs (Kumar et al., 2016). Direct *in vivo* administration of manipulated viral vehicle for gene delivery and *ex vivo* genetically engineered stem cells are the two principal approaches in advanced clinical gene therapy (Dunbar et al., 2018).

Over the last three decades, clinical gene therapy faced numerous obstacles and a great deal of failures, but it has now accomplished a huge progress in modern medicine and is finding its path into the clinic and the market (Corrigan-Curay et al., 2015), (Friedmann, 2007). In 2017, Luxurna, the first human gene therapy drug for an inherited retinal dystrophy, was approved by Food and Drug Administration () and entered the US market (Dias et al., 2017). In the same year, Kymriah and Yeskarta, two cell-based gene therapies for the treatment of acute lymphoblastic leukemia (ALL), were also approved by FDA (Butera, 2018; Vormittag et al., 2018). Various outstanding gene and cell-based gene therapies for both rare and common genetic disorders as well as life-threatening diseases, such as cancers and degenerative diseases, are in the evaluation phase prior to their translation into the clinic in the near future (Ehrke-Schulz et al., 2017; Colella et al., 2018). 2017 marks an important year of gene therapy and is considered as a launch point for a new era of modern gene therapy.

**Figure 1:**
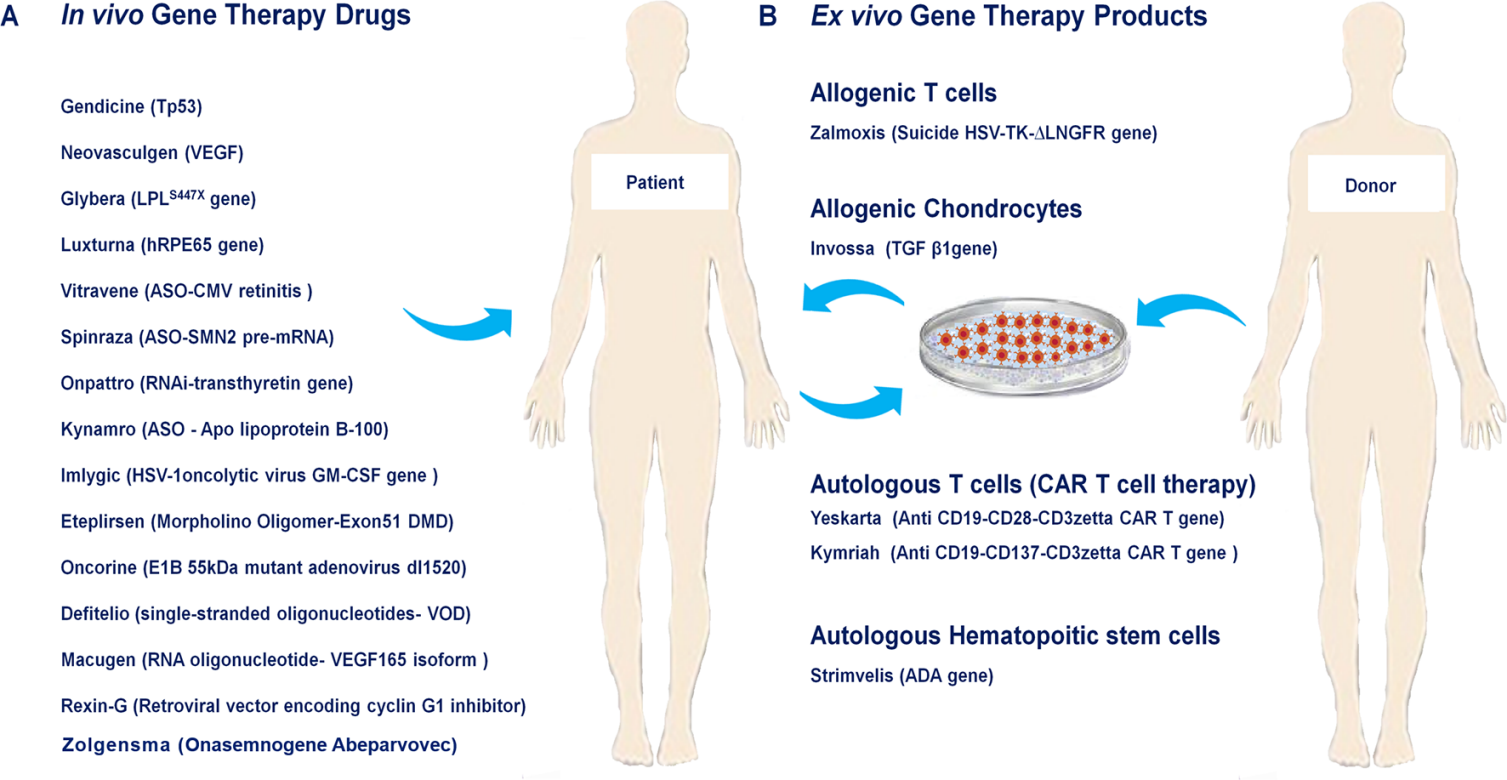
Approved human gene and cell-based gene therapy products. **(A)** *In vivo* approved gene therapy drugs such as Neovasculgen, Glybera, Defitelio, Rexin-G, Onpattro, Eteplirsen, Spinraza, Kynamro, Imlygic, Oncorine, Luxturna, Macugen, Gendicine, Vitravene as well as Zolgensma directly injected into their target tissue or organ. **(B)** *Ex vivo* gene therapy drugs include Zalmoxis as allogenic T cells, Invossaas allogenic chondrocytes, Yeskarta and Kymriahas autologous T cells (CAR T cell therapy), Strimvelisas autologous hematopoitic stem cells.

In the present review, we summarize the history of development, mechanism-of-action (MOA), target indications as well as primary clinical trials of the twenty so-far approved human gene and cell-based gene therapy products. Additionally, their limitation, safety, manufacturing, dosage and sales are discussed (**Figure 1**, **Table 1**).

**Table 1:** History and featured data of twenty approved gene and cell based gene therapy products.

Product name	Developer	Approval authority	Therapeutic indication and target tissue	Structure and mechanism of action	Dose	Limitations of use	Price 2018/2019	Clinical trials ID	Key publication
Fomivirsen (Vitravene)	Isis Pharmaceuticals with Novartis Ophthalmics	USA FDA (1998) EMEA (1999)	Treatment of CMV retinitis -eye	ASO -inhibition of immediate-early IE-2 protein	330 mg intravitreal injection on day 1 and day 15 of treatment for induction, then 330 mg monthly	Not recorded	withdrawn	NCT00002187 NCT00002156 NCT00002356 NCT00002355	Freedman (2001), Group (2002b), Group (2002c), Goldberg et al. (2005)
Gendicine	Shenzhen SiBionoGeneTech	China FDA (2003)	Head and neck squamous cell carcinoma (HNSCC)	Adenovirus contains wild-type p53.P53 production in cancer cells and inhibition of cell proliferation and induce apoptosis.	1.0 × 10^12^vector particles per vial	Women at pregnant or during breast-feeding period. Patients with generalized infection and fever of inflammation	$387 per injection	ChiCTR-TRC-08000094 ChiCTR-TRC-08000092 ChiCTR-TRC-08000091	Li et al. (2015) Zhang et al. (2018)
Pegaptanib (Macugen)	Eyetech Pharmaceuticals and Pfizer. Inc	USA FDA (2004)	Treatment of neovascular age-related macular degeneration - eye	Polynucleotide aptamer - inhibition VEGF165 isoform	0.3 mg once every 6 weeks by intravitreal injection	Need for repeated and frequent intravitreal injection	$765 per dose	NCT00858208 NCT01245387 NCT00788177 NCT00549055 NCT00460408 NCT00787319 NCT00312351 NCT00327470 NCT01712035	Gragoudas et al. (2004), D’Amico (2005), Battaglia Parodi et al. (2018)
H101 (Oncorine)	Shanghai Sunway Biotech	China FDA (2005)	Patients with last stage refractory nasopharyngeal cancer(Head and neck cancer), NSCLC lung cancer, Liver cancer, malignant pleural and peritoneal effusion and pancreatic cancer	E1B-55 kDa gene-deleted replication-selective adenovirus	Intratumoral injected at the dose of 1-3 vials per day, combined with 5-FU and cisplatin	Self-relieving local injection, mild to medium grade fever and influenza-like symptoms	–	NCT02579564	Nemunaitis et al. (2000) Xia et al. (2004)
Rexin-G	Epeius Biotechnologies	Philippine FDA (2007) USA FDA (2010)	metastatic solid tumours(pancreatic cancer, Breast cancer, Osteosarcoma, Sarcoma)	Retroviral vector contains cyclin G1. Causes cell death by blocking the cell cycle event in G1 phase and promoting apoptosis in cancer cells	5 × 10^9^ (cfu)/ml	Pregnant or nursing women Fertile patients	$5000 per bag 500,000 $ per treatment	NCT00505271 NCT00572130 NCT00505713 NCT00504998 NCT00121745	(Chawla et al., 2010) (Gordon and Hall, 2010)
Neovasculgen (Pl-VEGF165)	Human Stem Cells Institute	Russian Ministry of Healthcare (2012)	Atherosclerotic Peripheral Arterial Disease (PAD), including Critical Limb Ischemia (CLI) intramuscular transfer (calf muscles)	Plasmid DNA encoding the 165-amino-acid isoform of human vascular endothelial growth factor (pCMV - VEGF165)	1.2 mg per injection (totally 2.4 mg) In 2 ml injection water Each injection at 4-5 sites at calf muscles	Not recorded	$6600 for treatment course	NCT03068585 NCT02538705 NCT02369809	(Deev et al., 2015) Deev et al. (2015) Deev et al. (2017) Mäkinen et al. (2002)
Alipogenetiparvovec (Glybera)	Amsterdam Molecular Therapeutics	European Commission (EC) (2012)	Familial LPLD, Leg muscle	AAV1- contains the human lipoprotein lipase (LPL) gene variant LPL^S447X^	1×10^12^gc/kg	higher risk of bleeding and muscle disease in patients with immunodeficiency	over $1.2 m per patient	NCT02904772 NCT03293810	Bryant et al. (2013) Ferreira et al. (2014)
Mipomersen, (Kynamro)	ISIS Pharmaceuticals	USA FDA (2013)	Treatment of homozygous familial hypercholesterolemia - Liver, hepatocytes	ASO - Inhibition of the synthesis of apoB100	200 mg once weekly subcutaneous injection	It is not established in patients with hypercholesterolemia and without HoFH	$6910 For 1-ml vial	NCT00607373 NCT00280995 NCT00477594 NCT00694109	Santos et al. (2015)
Imlygic	Amgen	USA FDA (2016)	Multiple solid tumors (Melanoma, Pancreatic Cancer)	HSV-1oncolytic virus with deletions in the γ34.5 and α47 regions, which GM-CSF gene inserted into the deleted γ34.5 loci. Tumor Lysis and induce antitumor immune responses	10^6^-10^8^ PFU/ml	Pregnancy, Immunocompromised Patients	$65000 per treatment	NCT02014441 NCT01740297 NCT02366195 NCT00402025	Hu et al. (2006) Senzer et al. (2009)
Eteplirsen (Exondys 51)	Sarepta Therapeutics	USA FDA (2016)	Treatment of Duchenne muscular dystrophy -Striated muscles	PhosphomorpholidateMorpholino Oligomer (PMO) -Exon skipping	30 mg/kg once weekly by IV infusion	Not recorded	$1678 For 2 ml vial	NCT02420379 NCT01540409 NCT01396239 NCT00844597 NCT02286947 NCT03218995 NCT02255552 NCT00159250	Kinali et al. (2009) Cirak et al. (2011), Mendell et al. (2013), Geary et al. (2015)
Spinraza (nusinersen)	Biogen Ionis Pharmaceuticals, Inc.	USA FDA EMA (2016)	spinal muscular atrophy (SMA)/motor neurons and CNS	A modified 2’-O-2-methoxyethyl phosphorothioat ASO	12 mg intrathecally	Safe Lumbar puncture complications Intestinalproblerms Respiratory complications	$125000 per injection	NCT01494701 NCT01780246 NCT02052791 NCT01703988 NCT02292537 NCT02193074 NCT02594124	Finkel et al. (2016) Mercuri et al. (2017) Finkel et al. (2017) Bertini et al. (2017)
Defibrotide (defibrotide sodium)	Jazz Pharmaceuticals plc	USA FDA EMA (2016)	SOS/VOD with multi-organ dysfunction	Single-stranded oligodeoxyribonucleotides	4 doses per day (every 6 h) of 6.25 mg/kg 2-h Infusion	Hemorrhage Hypersensitivity Reactions Fertility, Pregnancy	$825 per 200-milligram or 2.5-milliliter vial	NCT00003966 NCT00628498 NCT00358501 NCT00272948	Corbacioglu et al. (2004, 2012) Richardson et al. (1998, 2010, 2016)
Luxturna (VoretigeneNeparvovec-rzyl)	Novartis Inc.	FDA (2017) EMA (2018)	Inherited Retinal Dystrophies (IRD), retinal pigment epithelial (RPE) cells	AAV2 is laboring a normal copy of the RPE65 gene	0.3 ml/eye	Conjunctival hyperemia, cataract, increased intraocular pressure and retinal tear, holes,and inflammation	$850000 or $425000 per eye	NCT00999609 NCT03602820	Miraldi et al. (2018) Russell et al. (2017)
Patisiran (Onpattro)	Alnylam Pharmaceuticals Inc.	FDA EMA (2018)	Familial amyloid polyneuropathy (FAP), Liver, Peripheral nerves Heart, Kidney, Gastrointestinal tract	dsRNA encapsulated in aliposome	0.3 mg/kg every 3 weeks (24 weeks)	Infusion-related reactions (IRRs)	$345000 per 2 mg/ml	NCT01559077 NCT01617967 NCT01961921 NCT01960348	Adams et al. (2018) Adams et al. (2017) Suhr et al. (2015)
Zolgensma (onasemnogene abeparvovec-xioi)	AveXis/ Novartis	USA FDA (2019)	Pediatric individuals less than 2 years of age diagnosed with SMA having bi-allelic mutations in SMN1 gene	Non-replicating rAAV9 containing cDNA of the human SMN gene under the control of CMV enhancer/chicken-β-actin-hybrid promoter	A sterile solution of 2.0 x 10e13 vector genomes (vg)/ml [1.1×10e14 vg/kg (body weight)] by intravenous infusion	The use of the dug in patients with advanced SMA Not recorded	Range ($2.125-5.0M/treatment), the average lifetime cost/patient is $4.2–6.6M	NCT03421977 NCT03505099 NCT02122952 NCT0330627 NCT03461289	AL-Zaidy et al. (2019), Rao et al. (2018), Dabbous et al. (2019), Waldrop and Kolb, (2019), Malone et al. (2019)
Strimvelis (GSK2696273)	GlaxoSmithKline (GSK)	EMA (2016)	ADA-SCID	Autologous HSC expressing ADA - retroviral vector transduced	2-20 million CD34+ cells per kg should be administered only once	Limitation of use in HCV infected patients (> 15 IU/ml nucleic acid test)	594,000 euros, or $648000 3 may 2017	NCT00598481	Cicalese et al. (2016) Aiuti et al. (2017) Cicalese et al. (2018)
Zalmoxis	MolMed SPAA	EMA (2016)	Hematopoietic Stem Cell Transplantation Graft vs Host Disease	Allogenic T cell expressing HSV-TK suicide gene- retroviral vector transduced	1 ± 0.2 x 107 cells/kg infusion 21-49 days from transplantation, in the absence of GVHD. repeated every month (max 4 times) to reach the ≥100 T cell count per µl.	Not used in children or T cell ≥100/µl in circulation	€149000 Dec 2017 €163900 EUR in Germany: ex-factory price	TK007 TK008	Denny et al. (2003) Ciceri et al. (2009) Vago et al. (2012)
Kymriah (Tisagenlecleucel CTL019)	Novartis Pharmaceuticals Corporation	USA FDA (2017)	Patients up to 25 years of age with B-cell precursor ALL that is refractory or in second or later relapse	Autologous CART cell targeting CD19- Lentiviral vector transduced	0.2-5 x 106 CAR-positive viable T cells per kg for <50kg patients or 0.1-2.5 x 108/kg for >50kg cases	lymphodepleting chemotherapy pretreatment is needed Not indicated for treatment of primary CNS lymphoma	$475000 Feb 2018	NCT02435849 NCT02445248	Schuster et al. (2017) Maude et al. (2018)
Yescarta (Axicabtageneciloleucel)	Kite Pharma, Incorporated	USA FDA (2017)	Non-Hodgkin lymphoma. B-cell lymphoma, high grade B-cell lymphoma, and DLBCL arising from follicular lymphoma	Autologous CAR T cell targeting CD19 -Retroviral vector transduced	2 × 106 CAR-positive viable T cells per kg	lymphodepleting chemotherapy pretreatment is needed Not indicated for treatment of primary CNS lymphoma	$373000 April 2018	NCT02348216	Roberts et al. (2017) Locke et al. (2017) Neelapu et al. (2017)
INVOSS (TissueGene-C or Tonogenchoncel-L)	TissueGene (now called KolonTissueGene)	South Korea’s Ministry of Food and Drug Safety (MFDS) (2017)	Kellgren-Lawrence Grade 3 knee osteoarthritis (OA) intra-articular space of the knee via X-ray guidance	Retrovirally transduced allogenicchondrocytes that upregulates TGF β1	1.8x10^7^ cells per 3 ml (mixture of transduced and non-transformed chondrocytes)	Peripheral edema arthralgia joint swelling Injection-sitepain	Not recorded	NCT01221441 NCT03412864	Cho et al. (2015) Cherian et al. (2015) Cho et al. (2017) Lee et al. (2018)

## Human Gene Therapy Products

### Vitravene (Fomivirsen)

Vitravene, also called as Fomivirsen is an antisense oligonucleotide (ASO) designed as a therapeutic strategy for cytomegalovirus (CMV) retinitis in HIV-positive patients who have not an option for CMV retinitis treatment (Azad et al., 1993; Anderson et al., 1996). Fomivirsen is the first ever gene-silencing antisense therapy approved for marketing by the FDA. This drug was developed through collaboration between Isis Pharmaceuticals and Novartis Ophthalmics and was approved by FDA in August of 1998 and 1 year later by EMEA (European Agency for the Evaluation of Medicinal Products) to treat cytomegalovirus retinitis (de Smet et al., 1999).

CMV retinitis, a serious viral eye infection of the retina in patients affected by AIDS, is estimated to affect 30% of these patients. Risk of AIDS-related CMV retinitis is almost always directly correlated with lower CD4 counts (less than 200 cells/µL) and lower peripheral-blood absolute CD4 lymphocyte counts (50 cells/µL or less) (Gallant et al., 1992; Studies of Ocular Complications of AIDS Research Group, 1992).

Fomivirsen is a 21-base phosphorothioate oligodeoxynucleotide which has a CpG motif near its 5’ terminal part. It specifically targets the IE-2 mRNA molecule which is encoding a protein required for CMV replication (Anderson et al., 1996; Mulamba et al., 1998). The recommended dose of Fomivirsen is 330 mg intravitreal injection on day 1 and day 15 of treatment for induction, then it is continued 330 mg every 4 weeks. The most frequently observed adverse effects include ocular inflammation and increased intraocular pressure. Half-life clearance of the drug is approximately 55 h in humans vitreous body (Group, 2002a; Uwaydat and Li, 2002).

In clinical studies by Vitravene Study Group, *lesion activity* regressed in 80% of participants and also it became completely inactive in 55% of participants during Fomivirsen therapy. Different studies indicate that Fomivirsen can successfully ameliorate the symptoms of CMV retinitis (Group, 2002a; Group, 2002b; Group, 2002c; Uwaydat and Li, 2002).

The development of highly active anti-retroviral therapy (HAART) significantly decreased the CMV retinitis incidence by 55–95%. Therefore marketing of Fomivirsen stopped in Europe and the USA in 2002 and 2006 respectively, as a consequence of the low demand. According to the Novartis Ophthalmics, demand for Vitravene was less than 100 units per year (Deayton et al., 2000; Varani et al., 2000; Kempen et al., 2003).

### Gendicine (rAd-p53)

Gendicine gene therapy drug is harboring Tp53 gene which has been developed to treat head and neck squamous cell carcinoma (HNSCC). This recombinant adenovirus was developed by Shenzhen SiBionoGeneTech and was approved by China Food and Drug Administration (*CFDA*) on October 16, 2003; and found its way to the commercial market in 2004 (Pearson et al., 2004; Peng, 2005).

Inactivation of different tumor suppressors including: TP53, NOTCH1, CDKN2A, PIK3CA and FBXW7 have been reported in HNSCC. Cells harboring those inactivated proteins could divide without control resulting in cell immortalization and increased risk of malignant transformation (Decker and Goldstein, 1982; Nikoo et al., 2017).

In Gendicine drug, E1 region of human serotype 5 adenovirus (Ad5) has been replaced by human wild-type Tp53. The expression of Tp53 in cancer cells stimulates antitumor properties by initiating apoptotic pathways, suppressing DNA repair and anti-apoptotic events as well as seizing the survival pathways. (Wilson, 2005). The vector is produced in HEK293 cells by co-transfection of the Tp53 expression cassette shuttle vector with an Ad5 genome recombinant plasmid. The cassette contains the Rous sarcoma virus promoter, the wild type human Tp53 gene and a bovine poly-A signal. Upon intratumor injection at a concentration of 1.0 × 10^12^ viral vector particles per vial, Gendicine binds to the coxsakievirus-adenovirus receptor and enters the tumor cells *via* receptor-mediated endocytosis, expressing ectopic Tp53 gene. The most common side effect with Gendicine is self-limiting fever of 37.5°C to 39.5°C which occurs usually 2 to 4 h after administration lasting for approximately 2 to 6 h (Chen et al., 2014; Li et al., 2015; Zhang et al., 2018).

The initial clinical trial of Gendicine drug was done in four hospitals of Beijing city between 1998 and 2003 years (Han et al., 2003; Wilson, 2005). Also, from 2003 to 2012, totally 16 human clinical studies were carried out for treatment of advanced stages and grades of head and neck cancer, malignant glioma, ovarian cancer, and hepatic cell carcinoma. Treatment with Gendicine resulted in a better overall response and higher survival rate compared to control groups (Chen et al., 2003, Zhang et al., 2003).

In a clinical study with patients suffering from nasopharyngeal cancer, administration of Gendicine in combination with radiotherapy resulted in higher survival rates compared to control groups (Zhang et al., 2005). A clinical trial reported that administration of Gendicine and chemotherapeutic drugs can significantly improve survival rate more than either chemotherapy-only or gene therapy-only groups in patients of advanced oral squamous cell carcinoma (Lu et al., 2004; Zhang et al., 2005).

### Macugen (Pegaptanib)

*Pegaptanib* was developed by *Eyetech Pharmaceuticals* and PfizerInc with brand name of Macugenis. It is a polynucleotide aptamer targeting vascular endothelial growth factor (VEGF165 isoform) for neovascular age-related macular degeneration (AMD) treatment. Pegaptanib was the first anti-angiogenic agent approved by the USA FDA in December 2004 and was the only therapy for treatment of AMD. It is also the first therapeutic aptamer with RNA structure achieving FDA market approval (Gragoudas et al., 2004).

AMD is the most common cause of severe vision loss and blindness among the aged individuals in the developed world. It is characterized by deterioration of the central part of the retina (Wong et al., 2014). Due to abnormal growth of blood vessels it accounts for 90% of severe vision losses (Fine et al., 2000). It has been suggested that VEGF plays a prominent role in growth and permeability of new vessels in AMD. Anti-VEGF agents are molecular therapies which attempt to block angiogenesis as well as vessel permeability (Friedman et al., 2004; Wong et al., 2008; Bressler, 2009; Solomon et al., 2014).

Pegaptanib is a 28-mer RNA oligonucleotide covalently linked to 2 branched 20-kD polyethylene glycol chains. It specifically binds to VEGF165 isoform at the heparin binding site; thus preventing its binding to VEGF receptors that are located on the vascular endothelial cells surface. VEGF165 has been implicated in pathological ocular neovascularization by increasing vascular permeability and inflammation (Ruckman et al., 1998; Robinson and Stringer, 2001). The recommended dose is 0.3 mg/90 µl of Pegaptanib administered once every 6 weeks by intravitreal injection into the eye. Based on preclinical data, Pegaptanib is metabolized by endo- and exonucleases and is not influenced by the cytochrome P450 system (Drolet et al., 2000; Vinores, 2003).

In two clinical trials involving totally 1186 participants, efficacy of Pegaptanib was determined by the ability of patients to lose less than 15 letters of visual acuity from baseline without dose–response. The result demonstrated that Pegaptanib is an effective therapy for AMD (Gragoudas et al., 2004; D’Amico, 2005). Moreover, a clinical trial assessed the side effects and the efficacy of Pegaptanib in the treatment of 23 participants suffering from neovascular AMD with previous history of arterial thromboembolic events (ATEs). Pegaptanib did not reveal any systemic or ocular side effects nor did it lead to any recurrent ATEs (Battaglia Parodi et al., 2018).

By 2010, sales of Pegaptanib declined due to better visual outcomes obtained through aflibercept, ranibizumab and bevacizumab drugs which are attested to be less expensive and appeared to be more effective than Pegaptanib (Brown et al., 2009; Sarwar et al., 2016). Nevertheless, Pegaptanib still holds a relatively small market share for the treatment of AMD. Currently, Pegaptanib is marketed by Bausch and Lomb and it approximately costs $765 per dose.

### Oncorine (rAd5-H101)

As the first oncolytic virus approved by CFDA, recombinant human adenovirus type 5 (rAd5-H101) was commercially marketed under the brand name of Oncorine in November 2005 and was manufactured by Shanghai Sunway Biotech. It was initially licensed for the treatment of patients with last-stage refractory nasopharyngeal cancer in combination with chemotherapy following phase III of the clinical trial (Liang, 2018).

Loss of Tp53 gene function is linked with resistance to chemotherapy and reduced survival rate of patients affected by non-small cell cancers of the breast, colon, lung, head and neck as well as ovaries. Therefore TP53 is considered as promising target for gene therapy of NSCC derived cancers. The E1B-55 KD gene has been totally depleted in Oncorine adenovirus which is responsible for p53 inactivation. Selectively, Oncorine propagates in P53-deficient cancer cells while the adenovirus which lacks the E1b-55KD fails to replicate in normal cells. Following cancer cell lysis, adenoviruses releases and infects neighboring cells initiating a cascade of Oncorine-mediated cell cytotoxicity (Lee et al., 2017).

The first Oncorine clinical trial was carried out on 37 participants with recurrent head and neck carcinoma testing intratumoral and peritumoral injection. The findings were in favor of highly selective tissue destruction, significant tumor regression and no toxicity evidence to injected normal peritumoral tissues (Nemunaitis et al., 2000). Oncorine in combination with chemotherapy in participants with late stages and high grades of different cancers, is administered by intratumor injection. The findings exhibited potential anti-tumor property to refractory aggressive tumors in combined with chemotherapy with minimal toxicity and accepted tolerance of participants (Lu et al., 2004). A phase III clinical study was carried out on 160 participants with head and neck or esophagus squamous cell cancers. Intratumoral injection of Oncorine was fallowed with cisplatin plus 5-fluorouracil (PF) or adriamycin plus 5-fluorouracil (AF) regimen versus PF or AF regimen alone. The result demonstrated a distinct efficacy and relatively safety of the drug (Xia et al., 2004).

Following the successful phase III clinical trial, the process of commercialization of Oncorine was launched and its efficacy and safety was examined on other cancer types including colon cancer and non-small cell lung cancer (NSCLC) (Reid et al., 2005). Oncorine is not basically associated with major adverse effects except for self-limiting reaction to local injection, mild to moderate grade fever and influenza-like symptoms.

### Rexin-G (Mx-dnG1)

Rexin-G is a retroviral vehicle harboring a cytocidal cyclin G1 construct and is considered the world’s first tumor targeting injectable gene therapy vector approved by FDA for metastatic pancreatic cancer. The drug was officially approved by Philippine FDA in December 2007 resulting in progression of clinical studies to Phase III trials in the USA (Gordon and Hall, 2010).

Rexin-G has a hybrid LTR promoter to express cyclin G1. The vector also comprises a neomycin resistance gene which is combined by the SV40 early promoter and is used for vector titer determination. Finally, the Rexin-G is produced by transient co-transfection of three separate vectors in 293T cells (Gordon et al., 2004; Gordon et al., 2006). Rexin-G triggers cell death and apoptosis (by suppressing the cell cycle in G1 phase) in cancer cells. Moreover, it is associated with neovasculature in preclinical studies (Gordon et al., 2006; Chawla et al., 2010).

The result from phase I/II trial of Rexin-G drug in participants with gemcitabine-resistant metastatic pancreatic cancer demonstrated it was well tolerated and safe. Also, elevated survival rate in patients was observed. Progressive clinical development of Rexin-G demonstrated the potential safety and efficacy of this gene therapy product for metastatic solid tumors that are resistant to standard chemotherapy (Gordon et al., 2006; Chawla et al., 2010).

### Neovasculgen (Pl-VEGF165)

In 2010, Human Stem Cell Institute of Russia developed Neovasculgen (PI-VEGF165), a plasmid DNA encoding VEGF 165 under the control of a CMV promoter for treatment of atherosclerotic Peripheral Arterial Disease (PAD). The drug was listed in Vital and Essential Drugs (EUVED) of Russian Ministry of Health in 2012 and was then distributed in the Russian market (Deev et al., 2015).

VEGF is a kind of angiogenic effector which triggers cellular proliferation and endothelial migration, angiogenesis as well as enhanced endothelial renovation. These events happen by inducing rapid secretion of nitric oxide and prostacyclin molecules from endothelium stimulating a vasculoprotective effect which is confirmed in some preclinical and clinical studies (Baumgartner et al., 1998; Mäkinen et al., 2002). Neovasculgen recombinant DNA is composed of a transcription start site, the encoding VEGF165 isoform, a polyadenylation signal, a splicing signal and SV40 transcription terminator (Bondar et al., 2015).

The main and only phase 2b/3 multicenter clinical study of Neovasculgen was conducted on 75 patients with PAD. The intramuscular administration of the drug resulted in an increase of pain-free walking distance as well as a significant increase in ankle-brachial index (ABI) and blood flow velocity (BFV). Thus, it was introduced as an effective therapeutic strategy of medium to severe claudication or limping due to chronic lower limb ischemia. (Deev et al., 2015).

Moreover, an international post marketing surveillance study confirmed the safety and efficacy of Neovasculgen in 210 patients with PAD suggesting the absence of no adverse effects (Deev et al., 2017). Authorization of the drug has recently begun in Ukraine, European Medicines Agency (EMA). However, FDA have not yet evaluated or validated the drug probably due to low penetrance of the disease. Neovasculgen would cost nearly *$*6,600 per treatment course.

### Glybera (Alipogenetiparvovec)

Alipogenetiparvovec, marketed as Glybera, is a gene therapy drug for Lipoprotein Lipase Deficiency (LPLD) treatment. It was developed by Amsterdam Molecular Therapeutics (AMT) in April 2012. In October 2012, the European Commission (EC) approved UniQure as a marketing authorization of Glybera for treating LPLD. Glybera is the first licensed gene therapy product for an inherited disorder in Europe (Bryant et al., 2013). However, given the lack of an appropriate relationship between supply and demand, it was declared a halt to Glybera marketing authorization in Europe in April 2017 (Hampson et al., 2017).

Familial LPLD is an autosomal recessive genetic disorder caused by loss-of-function mutations in LPL gene encoded lipoprotein lipase enzyme. Lack of the enzyme results in LPLD leading to improper digestion of certain fats and massive accumulation of fatty droplets (Faustinella et al., 1991). Glybera contains LPL gene variant cassette of LPL^S447X^ in a viral vector. The vector consisted of a protein shell part derived from adeno-associated virus serotype I (AAV1), the CMV promoter, a woodchuck hepatitis virus post-transcriptional regulatory element flanked by AAV2 derived inverted terminal repeats (ITRs). Furthermore, recombinant baculovirus technology was used for the Glybera production (Gaudet et al., 2012). Each vial of Glybera comprises of 3 × 1,012 genomic copies of alipogene tiparvovec (AAV1-LPL^S447X^) in 1 ml of a phosphate-based formulation buffer containing 5% sucrose (Bennett et al., 2016). Increased level of creatine kinase in the blood is observed following Glybera injection and it also increased the risk of bleeding and muscle disease in patients with immunodeficiency (Ferreira et al., 2014).

In a clinical trial, 22 subjects were recruited and injected with Glybera; 7 cases demonstrated a decreasing in median plasma triglyceride (TG) by at least 40% over 3 and 12 weeks involvement. The duration of Glybera efficacy did not rise by the immune suppression (Fine et al., 2000; Gaudet et al., 2013). However, in the high-dose cohort, VLDL, total cholesterol and TG content in the VLDL segments were increased at 12^th^ and 52^nd^ weeks post injection. Accordingly, there was a consistent rise in plasma LPL activity despite lack of prolonged effect on total plasma TG and chylomicron metabolism modifications (Andrew E Libby, 2013). In another clinical trial, there was a single group assignment which enrolled five patients with LPLD. TG concentrations decreased in 12 weeks upon treatment. Also, reduction in chylomicrons was observed (Robert Hettle et al., 2017). EC approval for Glybera was based on the results obtained from three Phase III clinical trials conducted in Canada and the Netherlands. Results from 27 patients with LPLD demonstrated that Glybera was well approved in all three clinical trials and no crucial safety signals were noticed. The one-time Glybera administration reduced the frequency of acute pancreatitis (Gaudet et al., 2016).

Glybera, one the highest-priced drug in the world, over $1.2 million per patient, has been withdrawn from the market because it has turned out to be a commercial loser. Some reporters declared that the drug has only been paid for once since its launch in 2017 (Friedman et al., 2004).

### Kynamro (Mipomersen)

Mipomersen, with a market name of Kynamro, is useful as an adjunct therapy for homozygous familial hypercholesterolemia (HoFH) (Raal et al., 2010; Mcgowan et al., 2012; Stein et al., 2012; Thomas et al., 2013). Mipomersen was developed by Ionis Pharmaceuticals as a novel ASO inhibitor for the cure of HoFH (Crooke et al., 2005). It was rejected by EMA in 2012 due to cardiovascular and liver adverse effects (Mahley, 2001). However, in January 2013, the USA FDA granted approval on its marketing as an orphan drug for the management of HoFH ().

FH is an autosomal dominant genetic disorder caused by mutations in genes of low-density lipoprotein receptor (LDL-R), apolipoprotein B (ApoB) and pro-protein convertase subtilisin/kexin type 9 (PCSK9). *APOB* gene encodes APOB protein that it is critical for LDL production and delivery (Goldberg et al., 2011; Raal and Santos, 2012; Hovingh et al., 2013).

Mipomersen is an ASO that interfere with the synthesis of ApoB. Mipomersen contains 20 nucleotides that binds to the coding region of the ApoB mRNA in a sequence-specific manner. Thus, it resulted in RNase H-mediated disruption of the mRNA molecule, thereby reducing synthesis of ApoB in the hepatocytes. Mipomersen decreases LDL-C, ApoB, total cholesterol (TC) and non-high- density lipoprotein cholesterol (non-HDL-C) in HoFH patients. Furthermore, Mipomersen causes a dose dependent decreasing of ApoB mRNA in the hepatocytes which is correlated with the reduction of ApoB -containing lipid particles in blood (Raal et al., 2010; Ricotta and Frishman, 2012).

Mipomersen is typically administered at a dose of 200 mg subcutaneously once per week. Around 85% of the drug is bound by Albumin in plasma. Half-life time of the injected drug is approximately 2 to 5 h. In addition, the half-life of the drug in plasma and tissue is approximately 1 to 2 months (Levin et al., 2007). Mipomersen is metabolized and processed initially by tissue endonucleases to generate shorter oligonucleotides available for further metabolism by exonucleases. Due to the risk of hepatotoxicity, Mipomersen is used with caution when prescribed with other LDL-lowering or hepatotoxic medications (Rosie et al., 2009; Crooke and Geary, 2013).

Raul et al. conducted four different phase III trials in various populations of FH patients. The outcomes demonstrated that Mipomersen invariably reduced plasma Lp(a) levels by the 28^th^ weeks by an average of 26.4% compared with the placebo groups (Santos et al., 2015). In phase III clinical trials, the most (84%) commonly-reported adverse effects were injection site reactions including erythema and pruritus, influenza-like symptoms (30%) such as fatigue, pyrexia and chills as well as nausea (14%) (Raal et al., 2010; Stein et al., 2012). In a phase III trial, cardiac events were observed with a high significant frequency in the Mipomersen group in comparison to the placebo (Mcgowan et al., 2012).

Due to the liver toxicity risk, Mipomersen is only used for patients under the constricted program called Kynamro^™^ Risk Evaluation and Mitigation Strategy. Single-use 1-ml vial of Mipomersen with a concentration of 200 mg/ml is available (Santos et al., 2015). The average price for 1 week of therapy with the drug is $6910. Nevertheless, due to adverse events, serious liver toxicity and reactions at injection site, a large proportion of patients discontinued the drug within 2 years.

### Imlygic (Talimogenelaherparepvec)

Imlygic or Talimogenelaherparepvec is a genetically manipulated oncolytic herpes simplex virus type 1 (HSV) that is developed to be used against multiple solid tumors such as unresectable cutaneous, subcutaneous and nodal lesions of melanoma (Chakradhar, 2017). Imlygic was created by BioVex Inc. under the brand name of OncoVEX^GM-CSF^. The drug was approved by the USA FDA in October 2015 for targeting melanoma. It was subsequently approved in Europe and Australia in 2016 (Reach, 2015; Printz, 2016; Chakradhar, 2017).

Imlygic is an advanced-generation of double-manipulated HSV-1 oncolytic virus with depletions in the γ34.5 and α47 segments, which has been replaced by the human granulocyte-macrophage colony-stimulating factor (GM-CSF) gene (Kohlhapp and Kaufman, 2016). The γ34.5 region deletion is mainly accounted for cancer-selective proliferation and reduction of pathogenicity. The γ34.5 gene plays a role in inhibiting protein synthesis of host cell upon viral infection. Thus suppressing γ34.5 seizes the virus replication in healthy cells. In cancer cells, γ34.5-deficient HSV-1 can still propagate (Johnson et al., 2015; Kohlhapp and Kaufman, 2016). The α47gene functions to antagonize the host cell transporter associated with antigen presentation. Consequently, the depletion of the gene results in reducing of MHC class I regulation and expression, which enhances the activities of antitumor immune responses. Moreover, two copies of the human GM-CSF gene are incorporated into the virus under the control of the CMV promoter providing high levels of gene expression. Local GM-CSF production by Imlygic is responsible for stimulating the immune system responses (Bommareddy et al., 2017).

Imlygic drug is sold as a sterile, preservative-free solution for intralesional injection, developed at a formal concentration of either 10^6^ or 10^8^ plaque-forming units (*PFU*)/ml. Patients receive the drug on days 1 and 15 of each 28-day period for 24 weeks intervention. Each dose should be injected into cutaneous, subcutaneous, and/or nodal lesions that are visible, palpable or detectable by ultrasound guidance. (Fukuhara et al., 2016).

Harboring a herpes virus, Imlygic could be reactive at a later time point, making herpes infections such as cold sores. Imlygic could cause more extensive medical conditions and side effects in patients with impaired immune system (e.g. HIV infected). (Puzanov et al., 2014; Fukuhara et al., 2016).

In several clinical trials, it has been observed that intralesional administration of Imlygic improves immunological response and causes regression in injected lesions (Hu et al., 2006). An analysis of phase II clinical trial outcomes revealed that Imlygic exerted a clear oncolytic effect on injected tumors as well as a secondary immune response as anti-tumor effect on non-injected lesions. Thus, it paved the path for initiation of the phase III of the drug (Senzer et al., 2009;Kaufman et al., 2010). In phase III trial, Imlygic significantly improved durable response rate versus GM-CSF in 436 patients with unresectable advanced-stage melanoma. (Andtbacka et al., 2015).

### Exondys 51 (Eteplirsen)

Eteplirsen, was developed by Sarepta Therapeutics under the *trade name of Exondys 51. This drug is* a 30-mer Phosphomorpholidate Morpholino Oligomer (PMO) designed to cause depletion of exon 51 of dystrophin gene. Expression of functional dystrophin protein in patients with duchene muscular dystrophy (DMD) who have mutated DMD gene is amenable by skipping exon 51 (Kole and Krieg, 2015; Stein, 2016). This group of patients cover approximately 13% of all DMD cases, making exon 51 a suitable target for gene targeting (Lim et al., 2017). *I*n September 2016, USA FDA approved Exondys 5 in an accelerated procedure based on the production of dystrophin in skeletal muscle found in some cases treated with the drug. (U.S. Food and Drug Administration, 2016; Andre et al., 2017)

DMD is a severe X-linked genetic disorder causing a degenerative muscle atrophy and early death. The worldwide incidence is estimated with 1/5000 male births (Mendell and Lloyd‐Puryear, 2013; Moat et al., 2013). The disease results from absence of the membrane-associated protein dystrophin, which renders a structural base connecting the cytoskeletal actin in muscle fibers to the extracellular matrix environment (Ervasti, 2007). The DMD gene encoding for dystrophin consists of 79 exons spread over 2.4 Mb region. Exon deletions that occurs more commonly in exons 47 to 63, derange the reading frame of the dystrophin mRNA molecule. This could result in losing protein synthesis in the striated muscle (Kinali et al., 2009).

Eteplirsen targets exon 51 in the dystrophin immature RNA (hnRNA) in DMD patients who carry a deletion between the terminus of exon 50 and the beginning of exon 52 comprising deletion of exons 45–50, 47–50, 48–50, 49–50, 50, 52 or 52–63. Then, the splicing machinery excludes the problematic exon from the final transcript, resulting in production of a shortened functional dystrophin protein (Kinali et al., 2009; Stein, 2016). Lack of charge and no interaction with nucleases have turned Eteplirsen into a highly stable and safe therapeutic drug. Lower protein interaction provides the drug insufficiently activate innate immune response (Summerton and Weller, 1997; Moulton, 2016).

*Exondys*51 was evaluated in two phase I/II, four phases II and two phase III clinical studies (Cirak et al., 2011; Burki et al., 2015; Geary et al., 2015; Mendell et al., 2016). Results from a confirmatory phase III trial comprising two 80-patient cohorts were required by the FDA to obtain the final approval. After 1 year of treatment, a significant average increase in dystrophin protein levels was seen at 0.22–0.32% of normal levels status. Taken together, clinical studies provided evidence that *Exondys*51 causes exon skipping, muscle cell penetration and induction of novel dystrophin synthesis. While *Exondys*51 is now available to applicants, further clinical trials are still required by the FDA to support the clinical benefit of the drug; 2 ml vial (50 mg/ml) of Exondys51 cost around $1,678 and the average price per patient of the drug stands at *$300,000* annually. (Andre et al., 2017)

### Spinraza (Nusinersen)

Nusinersen, commercialized under the name of Spinraza by Biogen, was the first ever medication approved for treatment of spinal muscular atrophy (SMA). Nusinersen was approved by USA FDA in December 2016 and by EMA in May 2017 (Garber, 2016).

SMA is a rare but one of the most common autosomal recessive disorder indicated by progressive disruption of motor neurons of the anterior horn of the spinal cord (Prakash, 2017). Deficiency in the survival motor neuron (SMN) protein is the molecular basis of the disorder (Lefebvre et al., 1995). As an evolutionarily-conserved protein, SMN is encoded by SMN1 and SMN2 genes and is critical for transcriptional regulation, telomerase regeneration and cellular trafficking of motoneurons (Singh et al., 2009). Deletion mutations (particularly in exon 7 and 8) in telomeric copies of the SMN1 gene have been observed in approximately 95% of SMA patients (Sun et al., 2005). According to the well-defined underlying mechanism of SMA, several genetic-based therapeutic procedures have been defined which primarily aimed to increase the accessibility of SMN protein in motor neurons (D’ydewalle and Sumner, 2015). These approaches included SMN1 gene replacement (Lowes et al., 2017), SMN2 alternative splicing modulation (Zanetta et al., 2014), SMN2 gene activation by previously approved drugs such as Salbutamol (Mercuri et al., 2016), Butyrates (Chang et al., 2001) and Valproic acid (Brichta et al., 2003), SMN stabilization (Mattis et al., 2009), neuroprotection (Kato et al., 2009), as well as stem cell-based therapies (Mercuri and Bertini, 2012).

Nusinersen (Spinraza) is an ASO which targets intron 7 on the SMN2 hnRNA (Castro and Iannaccone, 2014; Communes Internationales Des Substances, 2016), modulating alternative splicing by increasing inclusion of exon 7 in the final processed RNA. This results in higher levels of functional SMN protein in central nervous system (CNS) (Zanetta et al., 2014; Corey, 2017).

Nusinersen is administered intrathecal while performing lumbar punctures under direct supervision of a healthcare professional (Chiriboga, 2017). Following the administration of Nusinersen, it is distributed from the site of injection to motor neurons, vascular endothelial cells as well as glial cells in the CNS tissue (Finkel et al., 2016). The safety and/or tolerability profile of Nusinersen was acceptable in patients with SMA participating in several clinical trials (Finkel et al., 2016;Bertini et al., 2017;Finkel et al., 2017; Mercuri et al., 2017). Yet, the most frequently observed adverse effects of the drug were respiratory complications and elevated urine protein levels (Finkel et al., 2017). Moreover, intrathecal administration limits the treatment to CNS which is crucial for motoneurons but does not target other disorders in the heart, liver, pancreas, intestine and lung organs in SMA patients (Shababi et al., 2014). As such, an optimal treatment to restore SMN protein in peripheral tissues is also needed.

Several therapeutic phase I, II, and III clinical trials demonstrated promising findings and significant improvements in motor milestones (Hoy, 2017). Nusinersen was initially approved on 23 December, 2016 for treatment of the SMA in pediatric and adult patients in USA and is currently in the market. However, Spinraza is belongs to one of the most expensive drugs in the world with the price of $125000 per injection (Maharshi and Hasan, 2017). Although Spinraza expenses is supported by some health insurance providers in USA and France, Germany, Iceland, Italy and Japan, this expensive drug is not funded in other territories.

### Defitelio (Defibrotide)

Defibrotide, commercially known as Defitelio, is manufactured by Jazz Pharmaceuticals plc. Defitelio is a DNA derivative anticoagulant used for patients with hepatic sinusoidal obstruction syndrome/veno-occlusive disease (SOS/VOD) with renal or pulmonary dysfunction following the cytoreductive treatment prior to hematopoietic stem-cell transplantation (HSCT). The efficacy data coming from investigating 528 hepatic VOD participants with renal or pulmonary dysfunction following HSCT, supported approval of Defibrotide by USA FDA in March 2016 (Richardson et al., 2016). It was also evaluated and approved by EMA in May 2017.

SOS/VOD is an lethal indication of the conditioning regimens for HSCT which is distinguished by harmful hepatomegaly, hyperbilirubinemia, quick weight gain and gathering of ascitic fluid within the stomach (Bearman, 1995).VOD/SOS may also arise in patients treated by chemotherapy or calicheamicin–antibody drug conjugates (Mohty et al., 2015).

Defibrotide as the only approved gene therapy drug accessible for SOS/VOD patients with multi-organ damage (MOD) following HSCT has been associated with promising effects in the United States and the European Union. Defibrotide is a combination of primarily single-stranded oligo DNAs obtained from porcine mucosa tissue by controlled depolymerization with aptameric function on the vascular endothelial cells. It has antithrombotic, thrombolytic, anti-inflammatory and anti-ischemic properties (Francischetti et al., 2012). Defibrotide is considered an adenosine receptor agonist as it has affinity for receptors A1 and A2 on the plasma membranes of the vascular endothelium (Bianchi et al., 1993). It was originally introduced as therapeutic strategy for thrombophlebitis, and as prophylaxis of deep vein thrombosis (DVT) in Italy (Richardson et al., 2013).

Results of early clinical studies demonstrated promising response rates of 36–76% of Defibrotide in SOS/VOD with MOD (Richardson et al., 1998; Chopra et al., 2000; Corbacioglu et al., 2004; Richardson et al., 2010). Based on the result of a phase II clinical trial, 25 mg/kg/day was considered as the Defibrotide dosage with a base duration of 21 days (Richardson et al., 2010). Moreover, in a phase III trial, 356 pediatric participants at high risk of developing SOS/COD post-HSCT were involved to evaluate the prophylactic effects of Defibrotide. The result revealed a significant decreasing in SOS/VOD indication onset by Day +30 post-HSCT for the Defibrotide prophylaxis group in compared with control group (Corbacioglu et al., 2012).

However, several adverse side effects and reactions including coagulopathy, cerebral hemorrhage, hypotension, pulmonary hemorrhage, gastrointestinal hemorrhage and vomiting have been reported. Defibrotide was permitted marketing authorization by the EMA in October 2013 and FDA in March 2016 as the first approved therapeutic method for severe hepatic SOS/VOD post-HSCT indicated in adults and pediatric patients over 1 month of age. Defibrotide is an expensive DNA drug with a wholesale price of approximately $825 per 200mg or 2.5milliliter vial (daily price for the medicine is $7425 based on the recommended dose) (Chalandon et al., 2004; Strouse et al., 2016; Veenstra et al., 2017).

### Luxturna (Voretigene Neparvovec)

Voretigene Neparvovec-rzyl (AAV2-hRPE65v2), also called Luxturna which is developed and now available on the market by Spark Therapeutics. It is the first USA FDA-approved gene therapy drug for an inherited disease. Approval from FDA and EMA were granted on 19 December, 2017 and 23 November, 2018, respectively. Luxturna is applied intraocularly and is an orphan drug designated for the cure of inherited retinal dystrophy caused by bi-allelic RPE65 mutations (Ramlogan-Steel et al., 2018). This form of inherited retinal dystrophies (IRD), leads to clinical phenotypes of leber congenital amaurosis type 2 (LCA2) and retinitis pigmentosa type 20 (RP20). The most common form of IRD is retinitis pigmentosa (RP) with the reported impact of 1 in ∼4000 individuals. Both LCA2 and RP20 are inherited in an autosomal recessive way. Due to biallelic mutations in the RPE65 gene, its isomerase deficiency destroys the ability of retinal pigment epithelium (RPE) cells to react to the light. Finally, the accumulation of toxic precursors resulted in RPE cells death, progressive visual exacerbation and total blindness (Chung et al., 2018; Miraldi Utz et al., 2018).

Luxturna is applied by a subretinal injection following a vitrectomy where AAV2 targets RPE cells and brings in a normal copy of the RPE65 gene to compensate for the biallelic mutation. Resulting RPE65 protein acting as isomerohydrolase transforms the trans-retinyl esters to 11-cis-retinal, which is the natural ligand and chromophore of the opsins of rod and cones photoreceptors (Russell et al., 2018). In absence of functional RPE65, the opsins in not able to record light or transduce it into electrical responses to induce vision. Functional RPE65 protein resulted in the restoration of the visual cycle by regeneration of 11-cis-retinal (a critical visual pigment component) (Utsav Patel, 2018).

Safety and efficacy of Luxturna were examined in two clinical studies. Phase I trial was a dose-exploration safety study and phase III trial was an efficiency controlled study. 41 participants with mild to advanced vision loss at the time of the first administration took part in the clinical trial program. A statistically significant and clinically meaningful difference was observed in phase III clinical trial between patients (n = 21) and control groups (n = 10) in 1 year involvement. Over 100-fold improvement was revealed in the original patients group after 1 year. Evaluation by means of the bilateral multi-luminance mobility testing (MLMT) over the follow-up interval of minimum 1 year from the time of administration, determined that 93% (27 of 29) of all phase III trial patients took improvement in their vision function. The luxturna administration does not cause harmful immune responses. Spark announced a list price of $850,000 per patient, $425,000 per eye depending on the treatment (Russell et al., 2017; Dias et al., 2018;Russell et al., 2018).

### Patisiran (Onpattro)

With the brand *name of* Onpattro, Patisiran is the only FDA-approved RNA interference (RNAi) drug targeting polyneuropathy caused by hereditary transthyretin-mediated amyloidosis (hATTR) (Adams et al., 2018). The FDA approved this targeted RNA-based drug on August 10, 2018. Alnylam Pharmaceuticals, Inc. (Nasdaq), the leading RNAi therapeutics company, developed this lipid complex drug to treat familial amyloid polyneuropathy (FAP) in adults (Wood, 2018).

FAP, also known as hereditary transthyretinamyloidosis is caused by mutations in the gene encoding transthyretin (TTR), and is an autosomal dominant, progressive, multi systemic and life-threatening disease. In hereditary transthyretin amyloidosis, both wild and mutant-type transthyretin accumulate as amyloid in peripheral nerves, heart, kidney, and gastrointestinal tract giving rise to polyneuropathy and cardiomyopathy. Neuropathic alterations results in intense sensorimotor disruption with failure of daily life activities and ambulation (Escolano-Lozano et al., 2017; Plante-Bordeneuve, 2018).

Patisiran is a lipid nanoparticle containing an RNAi targeting the transthyretin mRNA. Once Patisiran enters the cell, transthyretin mRNA is cleaved by the RNAi leading to a decrease in circulating transthyretin protein. This reduces the amyloid accumulations which are linked to transthyretin-mediated amyloidosis (Butler et al., 2016). Administrated Patisiran targets primarily the liver. Nucleases cut Patisiran to nucleotides of various lengths (Suhr et al., 2015). Vitamin A deficiency is reported to be a main risk of Patisiran use by patients (Kerschen and Planté-Bordeneuve, 2016).

In an early clinical trial, abnormal transthyretin protein production rapidly diminished dose-dependently (Adams et al., 2016). Due to the phase II clinical trial results, Onpattro reduced the level of abnormal transthyretin protein by over 80%. The results also indicated that Patisiran improved neurological symptoms in all 27 patients for more than 24 months (Suhr et al., 2015). The outcomes of a phase III clinical trial revealed that Onpattro treatment lowered the abnormal transthyretin protein levels and improved FAP-related symptoms as well as the quality of life as compared with placebo groups (Adams et al., 2017).

### Zolgensma (Onasemnogene Abeparvovec)

Recently, AveXis a drugmaker owned by pharmaceutical giant Novartis, developed Onasemnogene Abeparvovec with the brand name of Zolgensma. It is the most recent authorization gene therapy drug by USA FDA (May 2019). It was previously well-known with compound name AVXS-101. Zolgensma is a proprietary gene therapy strategy for the cure of pediatric patients less than 2 years of age which have mutations in both alleles of *SMN1* gene. Zolgensma has been designed to render a healthy copy of SMN gene to seize disease progression through maintenance of SMN gene expression with a single, one-time intravenous infusion (Rao et al., 2018; Waldrop and Kolb, 2019; Malone et al., 2019; Mendell et al., 2017).

This drug is a non-replicating recombinant AAV9 containing a functional copy of human SMN1 gene under the control of CMV enhancer/chicken-β-actin-hybrid promoter (CB) to express SMN1 in motor neurons of SMA patients. The unique AAV9 capsid is capable to cross the blood-brain barrier allowing efficient CNS delivery by intravenous administration. The modification of the AAV ITR produces a self-complementary DNA molecule that forms a double-stranded transgene which enhances active transcription (Rao et al., 2018; Waldrop and Kolb, 2019).

The efficacy of Zolgensma in SMA patients with bi-allelic *SMN1* gene mutations was investigated in several clinical trials such as STR1VE (NCT03306277) and START (NCT02122952). Bi-allelic *SMN1* gene depletion, two wild type copies of the *SMN2* gene region, as well as lack of the c.859G > C mutation in exon 7 of *SMN2* gene was confirmed in all participants. 21 patients (mean age of 3.9 months) enrolled at the ongoing clinical trial of STR1VE. All the patients received 1.1 × 10[1][4] vg/kg of Zolgensma drug. Comparing results from the clinical study to accessible natural history data of participants with infantile-onset SMA renders initial evidence of the effectiveness of Zolgensma. The next clinical trial involving 15 patients was named START and involved a low-dose cohort of 3 patients with the mean age of 6.3 months and 12 patients in a high-dose cohort with the mean age of 3.4 months. The low-dose cohort received approximately one-third of the dosage of drug as the high-dose cohort. Comparing results from low- and high-dose cohorts showed a dose-response relationship that provides the clinical use of Zolgensma. (AL-Zaidy et al., 2019; Dabbous et al., 2019)

Elevated liver enzyme of aminotransferases has been reported with Zolgensma therapy. For patients with impaired liver function it is recommended to examine hepatic aminotransferases [aspartate aminotransferase (AST) and alanine aminotransferase (ALT)], total bilirubin and prothrombin before drug infusion. Transient decrease in platelet counts (considered as thrombocytopenia criteria) were revealed at different time points after Zolgensma infusion. Thus, monitoring platelet counts before Zolgensma use and on a regular basis eventually is recommended. Also, transient elevated cardiac troponin-I levels were reported following drug infusion in clinical studies. However, the clinical value of these findings is still unclear (Malone et al., 2019).

The drug also carries a heavy price tag of more than $2.125 million for a one-time treatment which it is the most expensive gene therapy on the market, yet relative cost-effective.

## Human Cell-Based Gene Therapy Products

### Strimvelis (GSK-2696273)

Adenosine deaminase (ADA) deficiency is considered as an autosomal recessive genetic disease causing the severe combined immune deficiency (SCID). ADA enzyme deficiency is the most widespread kind of the SCID accounting for 15% of all patients (Hershfield, 2009). Affected Patients suffer from different metabolic disorders and life-threatening opportunistic infections caused by severe immune deficiency and lymphopenia (Whitmore and Gaspar, 2016). Current therapeutic approaches consist of hematopoietic stem cell transplantation (HSCT), Enzyme replacement therapy (ERT) as well as gene therapy tool.

HSCT from a matched sibling donor will be curative by permanently increasing the overall survival (86%) (Hassan et al., 2012); however, such a donor is available for about 30% of the patients (Tiercy, 2016). In the case of matched unrelated (66%) or haploidentical (43%) donors, less survival will be achieved (Hassan et al., 2012). The other treatment choice is ERT with PEG-ADA which can offer disease relief to the patients but is not a curative method and requires multiple administrations (Booth and Gaspar, 2009). Gene therapy has provided promising treatments for patients affected by ADA-SCID. Gene therapy attempts for ADA-SCID treatment started in early 1990, when the first gene therapeutic trial was carried out at the NIH Clinical Center using gamma-retroviral mediated gene delivery to the autologous peripheral blood lymphocytes of a 4-year-old ADA-SCID affected girl (Ferrua and Aiuti, 2017).

In 2016, the EC approved the GlaxoSmithKline (GSK) stem cell based *ex vivo* gene therapy as a therapeutic option for ADA-SCID. The gene therapy product, also named Strimvelis (GSK2696273), was initially evolved by the San Raffaele Telethon Institute. The manufacturing procedure from HSC transduction to the medicine infusion requires expertise and high standards in product management and, for the time being, is only administered at the San Raffaele Hospital in Milan (Aiuti et al., 2017). Strimvelis consists of autologous hematopoietic stem/progenitor (CD34+) enriched cells transduced *ex vivo* with a retroviral delivery system to express the functional human adenosine deaminase (ADA) cDNA sequence which can replace the enzyme deficiency. At least 4 million purified CD34+ cells/per/kg are required to produce Strimvelis and it is indicated to treat ADA-SCID patients without matched related donor. According to the manufacturer’s recommendation, the optimum dose range of Strimvelis (2-20 million CD34+ cells/per/kg) should be administered only once to achieve the best outcome (updated 08/06/2016). Prior to autologous infusion of the gene-transduced CD34+ cell, pre-conditioning with low dose Busulfan (an anti-neoplastic alkylating agent) is necessary (Cicalese et al., 2016). The CD34+ enriched cells will be transduced with retroviral vectors encoding the human ADA cDNA and similar to other retroviral-based gene therapy methods, the potential integration mediated mutagenesis needs to be considered (Cicalese et al., 2016).

In a phase I/II trial, Strimvelis drug was evaluated on totally 18 ADA‐SCID children. Evidence of increased immunoglobin production and increased T cell subtypes (CD3+, CD4+ and CD8+) indicated that Strimvelis infusion can promote both cellular and humoral immunity. Gene modified blood cells were stably present in circulation of treated patients in the post gene therapy phase. Increased ADA enzyme activity and consequent decline in dAXP (metabolite accumulated in AD deficiency) demonstrated the gene therapy engraftment in treated patients (Cicalese et al., 2016). Following gene therapy, the most commonly observed adverse effects were upper respiratory infections, gastroenteritis as well as rhinitis among the others, with the highest incidence occurring over the time span between pre-treatment up to 3 months into the treatment. Fortunately, no leukemic transformation was observed in the following 13 years (Cicalese et al., 2018).

### Zalmoxis (Allogenic T cells encoding LNGFR and HSV-TK)

The allogenic HSCT remains the main therapeutic option for the high risk hematopoietic malignancies. Meanwhile, in the case of haploidentical HSCT, it may end up in failure due to Graft Versus Host Disease (GVHD) (Lorentino et al., 2017). To prevent GVHD in haploidentical HSCT, one option is T cell depletion prior to transplantation which may lead to poor survival due to a severe delay in immune reconstitution (Ciceri et al., 2009). The MolMedproduct, called Zalmoxis, made it possible to overcome this limitation. The partially-matched donor T cells will be modulated genetically to express HSV-TK (thymidine kinase enzyme) as an inducible suicide gene. In the case of GVHD initiation, the engineered transplanted T cells can be targeted and killed *via* administration of the pro-drug Ganciclovir (GCV), which is activated to a toxic triphosphate form by HSV-TK enzyme (Denny, 2003).

Zalmoxis contains genetically-modified allogeneic T cells using a retroviral delivery system expressing a shortened human low affinity nerve growth factor receptor (ΔLNGFR) and HSV-TK Mut2 to transduce the allogeneic T immune cells. ΔLNGFR expression cassette was used as the selection marker of the transduced manipulated T cells and the HSV-TK Mut2 expression provides the suicide gene induction if necessary. Infusion of the genetically manipulated donor T cells to HSCT (T cell depleted) transplant patients can simply reconstitute the immunity to protect from infections and confront cancer cells; however, donor cells can potentially target the host cells leading to GVHD. In this case, suicide gene induction by GCV administration can kill the donor T cells expressing HSV-TK and GVHD control (Mohty et al., 2016; Mullard, 2017).

The producer recommended the dose of 1 ± 0.2 × 10^7^ cells/kg for the infusion, following 21-49 days after transplantation, without GVHD. The infusion should be repeated monthly up to 4 months to reach the ≥100 T cell count/µl. The Zalmoxis is not allowed to be administrated in participants younger than 18 years age or in the case of T cell ≥100/µl in circulation (updated 5/09/2016).

The application of the Zalmoxis as adjutant treatment of leukemia patients in T cell depleted haploidentical stem-cell transplantation was evaluated in a phase I–II clinical trial (NCT00423124). Briefly, totally 28 participants were infused serially with 0.9-40 × 10^6^ cells/kg of genetically modified purified donor T cells expressing TK after transplantation monthly up to a maximal point of four infusions. As many as 22 participants obtained immune reconstitution (CD3 + counts ≥100 cells/μl) 23 days (13–42) following the infusion, wile10 ones evolved acute GVHD (grade I–IV) while one was reported to have chronic GVHD, which was managed by suicide gene induction. The most common adverse effect of TK cell infusion was acute GVHD. Altogether; these findings indicated that Zalmoxis can enhance the immune system reactivation following T cell depleted HSCT, with controllable GVHD (Ciceri et al., 2009).

Engraftment of the TK positive allogenic donor T cell can activate the host thymopoesis with elevated systemic IL-7 (early maturation of T-cell cytokine), providing a high number of newly generated TK-negative naive lymphocytes. This finding suggests that even after suicide gene induction and TK-positive T cell elimination to control GVHD, long-term immune reconstitution will reduce the risk of infection after HSCT (no infection was recorded in treated patient of TK007 after 166 days) (Vago et al., 2012). Consistently, results published from the phase III clinical trials (TK008, NCT00914628) carried out in Europe and United States revealed that infusion of genetically engineered allogenic T cells (MM-TK) (1*107/kg up to four monthly infusions) can increase the 1-year-disease-free survival by 22% in patients (30% vs 52%) (Bonini et al., 2015).

Analysis of 36 individuals treated with Zalmoxis in different trials (22 patients from TK007 trial and 14 patients from the ongoing phase III TK008 trial) and 127 control patients, demonstrated 1 year overall survival (OS) (40% vs 51%, p = 0.03) in those patients who survived relapse-free 3 weeks post transplantation. Nevertheless, the relapse probability and leukemia free survival were not significantly different(updated 5/09/2016).

Zalmoxis can provide promising curative improvements for HSCT patients when the matched donor is not available. It can benefit the patients in different aspects including post-transplant GvHD control, Graft versus Leukemia (GvL) improvement and relapse decrease as well as more important long term immune reconstitution leading to reduced infection probability and mortality.

### Kymriah (Tisagenlecleucel)

In 2012, Novartis drugmaker, in collaboration with the University of Pennsylvania, began to study chimeric antigen receptor T-cell (CAR T) therapies for acute lymphoblastic leukemia (ALL) treatment. The first approved CAR T-cell-based gene therapy by United States FDA (August 2017) was kymriah (tisagenlecleucel, Novartis Pharmaceuticals, co), which is used for the treatment of children and young adult patients (up to 25 years old) with relapsed B-cell ALL. ALL is the most common malignant tumor diagnosed in children (Belson et al., 2007) and the second most common acute leukemia in adults (Terwilliger and Abdul-Hay, 2017). Despite the advances in common therapeutics of chemotherapy or stem cell transplantation, the relapsed or refractory leukemia still remains a clinical challenge (Kell, 2016).

CAR is an engineered receptor exposing extracellular cancer-specific epitopes (scfv region) linked to the transmembrane and intracellular TCR derived and stimulatory domains. Four generations of CAR T-cells have been developed up to now with different cancer-killing efficiencies and cytokine releasing abilities (Smith et al., 2016). The scfv antibody domain connects to the target antigen in an MHC independent manner leading to CAR clustering and activation of T-cell through intracellular region composing the TCR-derived CD3ζ chain, without (first generation) or with co-stimulatory domains of the CD28 (generations 3 and 4), OX40/4-1BB (TNF/NGF family, generation 3). The 4th generation of CART proves more effective in solid tumors elimination, also known as TRUCK cells which are induced for secretion of cytokines (IL-12) in target tissue for augmentation of the t cell response and host innate immunity activation for elimination of antigen negative cancer cells (Chmielewski et al., 2014; Chmielewski and Abken, 2015). The clinical efficacy depends on the durable persistence of CAR T-cells assured by co-stimulatory signals protecting the CAR T-cells from ‘activation induced cell death’ (long term). More importantly, the activated CAR T-cells render target-specific memory cells which it induces inhibiting tumor relapse. The CAR T-cell has been clinically used in leukemia and lymphoma (Hartmann et al., 2017); this is while, clinical trials have shown that it is also applicable to kill solid tumors (Rodriguez et al., 2017). While enjoying approved clinical efficacy, CAR T-cells therapy causes side effects including on-target off-organ toxicity, cytokine release syndrome (CRS), neurotoxicity, auto-reactivity and other common reactions associated with antibody therapeutic like fever, nausea, and hypotension (Abken, 2015).

Kymriah is composed of autologous T cells suspension that are genetically manipulated with a lentiviral delivery system to produce a CAR comprising of a murine single-chain antibody fragment (scFv) specific for CD19 joint to an intracellular cytoplasmic domain for 4-1BB (CD137) and CD3 zeta with a CD8 transmembrane hinge. The autologous T cells is transduced with a self-inactivating lentiviral vector pseudo-typed with a VSV-G envelope derived from HIV-1 genome in ex-vivo condition. The vector will be integrated into the genome of transduced cells and will result in production of the tisagenlecleucel CAR under the regulation of a constitutively active promoter. After binding to target cells (CD19 expressing cells), the activated tisagenlecleucel CAR will initiate the antitumor activity through CD3 domain. The intracellular 4-1BB costimulatory domain will augment the antitumor reaction and also ensures durable persistence of the CAR T-cells (Updated: 05/07/2018; U.S. Food and Drug Administration, 2017).

As part of safety concerns to avoid replication competent retrovirus (RCR) during Kymriah manufacturing, all needed HIV-1 helper sequences and the pseudo-typed VSV-G envelope sequences are distributed among different constructs with minimum sequence homology, which ensures limited RCR. Moreover, the RCR formation will be checked in the peripheral blood samples of treated patients using VSV-G qPCR (U.S. Food and Drug Administration, 2017). The recommended dosage depends on body weight: 0.2-5 x 10^6^ CAR-positive viable T cells per kg for <50kg individuals or 0.1-2.5 x 10^8^ per kg for >50kg cases. Similar to other CAR T products, it should be infused after completion of the lympho-depleting chemotherapy (Updated: 05/07/2018).

In a phase II trial, the efficacy of Kymriah was evaluated in 63 pediatric or young patients with relapsed B-cell ALL. The results showed that 81% of patients represent overall remission with no minimal residual disease. The remission was durable with overall survival of 90% for 6 months and 76% after 1 year. The Kymriah was persisted in patients’ blood as long as 20 months. The cytokine release syndrome was observed in 77% of patients (Maude et al., 2018). Kymriah was successful in treatment of adult patients with diffused large B-cell Lymphoma (DLBCL), the dominant form of lymphoma. In this global phase II trial, as many as 99 patients (22-76 years old) were infused with Kymriah, formerly CTL019, which resulted in 95% of complete remission in 3 months, sustained at 6 months. The CTL019 was persisted in blood of patients for up to 367 days and CRS was observed in 58% of treated patients (Schuster et al., 2017).

### Yescarta (AxicabtageneCiloleucel)

In October 2017, Yeskarta (Axicabtageneciloleucel, Axi-Cel, Kite Pharma, Inc), another CAR T-cell therapy was approved by USA FDA for the treatment of adult patients suffering from aggressive non-Hodgkin lymphoma with the history of at least two failed systemic therapies. DLBCL is the dominant subtype of blood malignancies with a wide range of clinical and genetic heterogeneity (Belson et al., 2007). The majority of DLBCL new cases respond to a standard care therapy consisting of rituximab and chemotherapy; however, approximately 10–15% of them experience a refractory state (Roberts et al., 2017). According the FDA, Yescarta can be administrated in the case of DLBCL, primary mediastinal large B-cell lymphoma, high-grade B-cell lymphoma and DLBCL resulting from follicular lymphoma (Updated 02/20/2018).

Yescarta is a CD19-directed ex-vivo modulated autologous T cells transfected with gamma-retroviral vector. It expresses a CAR consisting of an extracellular murine anti-CD19 single-chain variable fragment fused to a cytoplasmic domain comprising of CD28 and CD3-zeta co-stimulatory domains. The autologous T-cells harvested from patients by leukapheresis would be shipped to legal centers. The transferred T-cells are then enriched in a closed system in the Yeskarta manufacturing center. The activated T-cells (by anti-CD3 and IL-2 treatment) would be transduced with a retroviral vehicle expressing the anti-CD19 CAR gene. Finally, following less than 10 days for manufacturing processes, the Yeskarta CAR T-cell is ready for infusion back into the patient (Roberts et al., 2017).

The genetically manipulated autologous CAR T cells can target and eliminate CD19-positive cells when infused back into the patient. 2 × 10^6^ CAR-positive viable T cells per kg body weight is recommended dose which is administrated following a lymphodepleting chemotherapy. It is not designate for the cure of patients with primary CNS lymphoma (Updated 02/20/2018).

In a phase II trial, 101 patients received Yeskarta and the result indicated an 82% objective response rate and a 54% complete response rate with a 52% overall rate of survival within 18 months. The common observed side effects were neutropenia (78%) and anemia (43%) (Locke et al., 2017; Neelapu et al., 2017). The Zuma-1 results showed approximately six-fold higher complete remission rates when compared to the achievements of the SCHOLAR-1, the benchmark multi-cohort retrospective study on refractory DLBCL patients outcomes (Neelapu et al., 2016).

### Invossa (TissueGene-C)

Invossa (TissueGene-C) has completed phase III trials in the USA and attained marketing approval in Korea by KolonTissueGene as a first-in-class cell mediated gene therapy strategy for the treatment of symptomatic and persistent knee osteoarthritis (OA). It contains 3:1 mixture ratio of non-transformed and retrovirally transduced allogenic chondrocytes that upregulate transforming growth factor β1 (TGF β1) (Lee, 2018).

Since the beginning of 2015, application of the Invossa progressed rapidly through its clinical trials and commercialization in the USA. To assess the safety and efficacy of Invossa, several phase II and III clinical trials and also post marketing surveillance studies were completed or recruited in Korea and USA. Studies showed that Invossa treatment can improve knee OA significantly (Cherian et al., 2015). In another phase II clinical trial on patients with a confirmed diagnosis of knee OA, Invossa treatment improved pain, sport activities and quality of daily life (Cho et al., 2015). Additionally, in the randomized double blind, multi-center, placebo-controlled phase III trial on 156 patients with confirmed knee OA, Invossa treatment successfully improved the quality of patients’ lives (Cho et al., 2017). In the most recent phase III clinical trial on 163 OA patients, Invossa significantly improved the function and pain relief with structural refinement and mild to severe adverse effects. The most frequent side effects in the Invossa are reported as peripheral edema, arthralgia, joint swelling, and injection-site pain (Lee, 2018).

Finally, KolonTissueGene achieved medical product approval of Invossa in July 2017. A post-marketing surveillance of Invossa injection in patients with OA is now involving to evaluate the safety and effectiveness of this new drug. What’s more, several studies have investigated the underlying molecular mechanisms in the effectiveness of Invossa on OA. The majority of studies suggest that Invossa provides an anti-inflammatory surroundings in the arthritic knee joints by means of macrophage polarization which is crucial for modifying the disease (Choi et al., 2017; Lee et al., 2018).

### Timeline and the Future Trend of Gene Therapy Market

**Figure 2:**
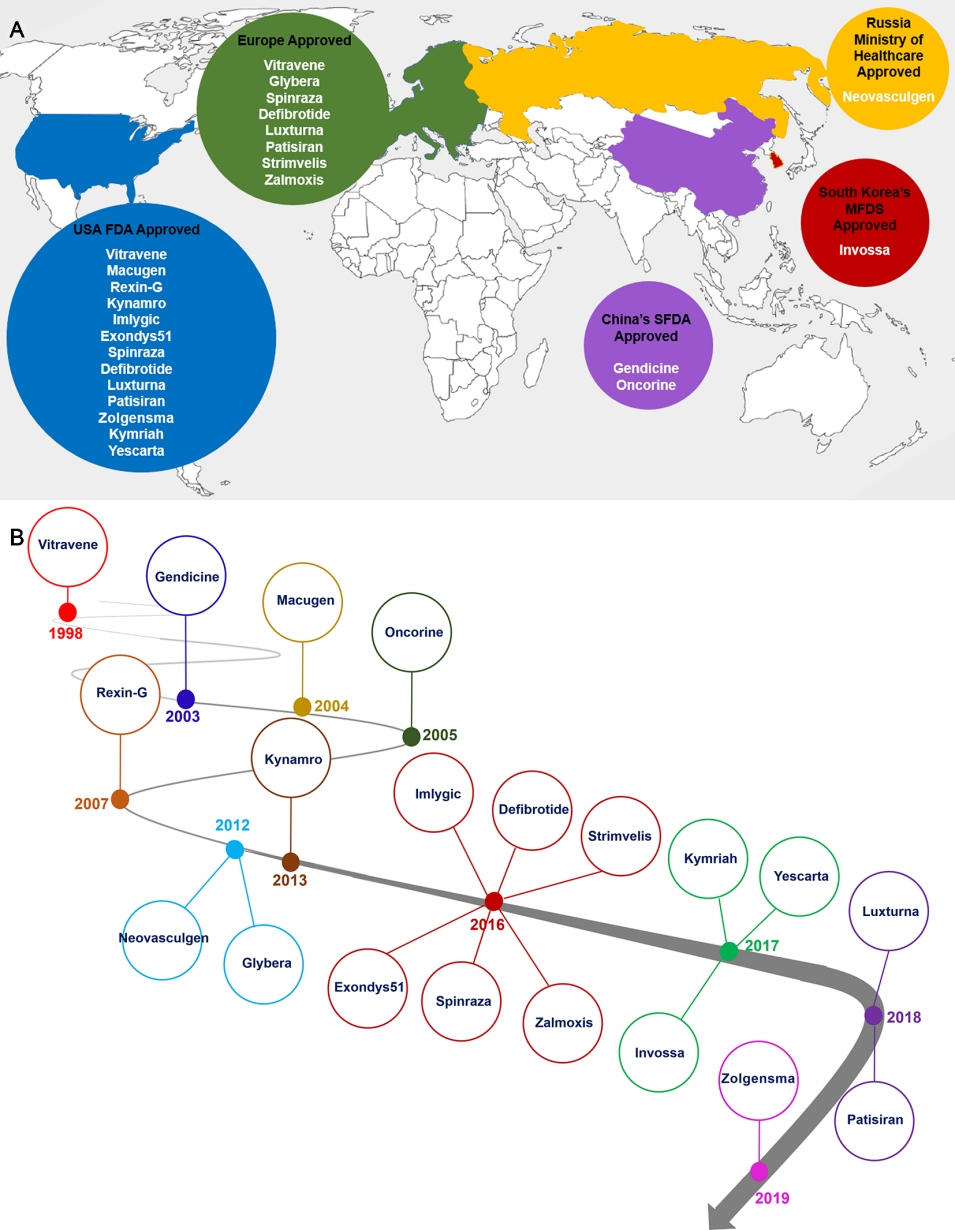
Distribution countries of approval gene therapy drugs **(A)** and timeline of gene therapy development **(B)**. As it is shown the North America and Europe are main parts of the gene therapy development and market **(A)**. Gene therapy market began with approval of Vitravene drug in 1998, and it was continued with approval of Zolgensma drug in 24 May, 2019. 2016 and 2017 years were promising points in gene therapy market since near 10 gene therapy products such as Imlygic, Defibrotide, Spinraza, Zalmoxis, Exondys51, Strimvelis, Invossa, Yeskarta and Kymriah were approved by relevant authority **(B)**.

In 1998, Vitravene was the first clinic antisense gene therapy product for the treatment of CMV retinitis in HIV-infected patients that was approved by USA FDA (de Smet et al., 1999). For targeting HNSCC, a common form of cancer in China, Gendicine was approved in 2003. It is a recombinant human adenovirus expressing Tp53 gene, which entered the Chinese market in 2004 (Pearson et al., 2004; Peng, 2005). Also in 2004, Macugen was approved by USA FDA as the first therapeutic RNA treatment of AMD (Gragoudas et al., 2004). Oncorine is the second approved gene therapy drug in China which is used for cancer treatment (Liang, 2018). The next gene therapy drug targeting pancreas cancer is Rexin-G which was approved by USA FDA in 2007 (Gordon and Hall, 2010). Neovasculgen gene therapy drug is only developed and approved for Russian market in 2012 (Deev et al., 2015). Europe approved their first gene therapy drug, Glybera, in 2012 to treat LPLD. Glybera is nowadays not anymore listed on its developer company product pipeline (Hampson et al., 2017). Kynamro is another antisense drug inhibiting apoB in HoFH patients which was approved by USA FDA in 2013 (Raal et al., 2010; Mcgowan et al., 2012; Stein et al., 2012; Thomas et al., 2013). Imlygic was approved by the USA FDA for targeting melanoma in 2015 (Reach, 2015; Printz, 2016; Chakradhar, 2017). As the tenth clinical gene therapy product, Exondys51 was approved by USA FDA in 2016 for treatment of DMD patients (U.S Food and Drug Administration, 2016; Andre et al., 2017). Spinraza as the first gene therapy to treat SMA patients was approved by USA FDA in 2016 and by EMA in 2017 (Garber, 2016). Defibrotide was developed to treat patients with hepatic SOS/VOD prior to HSCT and was approved by USA FDA in March 2016 (Richardson et al., 2016). In 2016, Strimvelis, was approved in Europe a gene therapy for treating ADA-SCID patients. 2017 was promising year for stem cell based gene therapy (Hershfield, 2009), (Whitmore and Gaspar, 2016). The United States finally approved *ex vivo* CAR-T therapies. Kymriah is considered the first CAR-T therapy targeting ALL which was approved by USA FDA in August of 2017. As second CAR-T therapy product, Yescarta, treating adult patients with diffuse large B-cell lymphoma, was approved by USA FDA in October of 2017 (Roberts et al., 2017; Terwilliger and Abdul-Hay, 2017). Zalmoxis and Invossa are stem cells based gene therapy products than entered the clinic in 2016 and 2017 respectively (Lorentino et al., 2017) (Lee, 2018). Spark Therapeutics has been granted an FDA approval for their Luxturna gene therapy drug in 2018. This pharma company utilizes an AAV system to deliver RPE65 gene into the patients eye suffering from retinal dystrophy caused by RPE mutations (Ramlogan-Steel et al., 2018). *As the first RNAi* drug, Patisiran was approved by FDA in 2018 (Adams et al., 2018). In May 2019, Zolgensma was approved by USA FDA as the twentieth approved gene therapy products until now (**Figure 2**). Finally, at the beginning of 2019, Zaynteglo, also known as Lentiglobin got conditional approval by EMA for beta thalassemia. Furthermore, BMN 270 a drug for hemophilia A, and GT-AADC a product for AADC deficiency are two highlighted gene therapy products that may be approved until 2020.

**Table 2** summarized more gene therapy clinical trials that are currently ongoing for advanced stages of trials and/or approval.

**Table 2:** Highlighted ongoing gene therapy products.

Product name	Developer/ sponsor	Structure and mechanism of action	Therapeutic indication and target tissue	Clinical trials ID
Zynteglo (Lentiglobin BB305)	Bluebird Bio	*Ex vivo* transplantation of hematopoietic stem cells manipulated by a lentiviral vehicle transferring HBB	Beta thalassemia (*conditional approved by EMA*)	NCT01745120 NCT02151526 NCT03207009 NCT02140554 NCT02906202 NCT03207009
BMN 270 (Valoctocogene roxaparvovec)	BioMarin Pharmaceutical	An AAV vector transferring factor VIII in hemophilia A individuals with Residual FVIII Levels ≤ 1 IU/dl	Hemophilia A	NCT03392974 NCT03370913 NCT02576795 NCT03520712
AAV2-hAADC	PTC Therapeutics	AAV serotype 2 expressing human AADC (hAADC)	Aromatic L-amino Acid Decarboxylase (AADC9 deficiency AADC	NCT02926066 NCT02852213 NCT02399761 NCT02926066 NCT01395641
Lenti-D	Bluebird Bio	*Ex vivo* Transplantation of CD34+ stem cells manipulated by lentiviral vector to contain a functional ABCD1 gene	Cerebral adrenoleukodystrophy (CALD), ABCD1 gene	NCT03852498 NCT01896102 NCT02698579
NSR-REP1 (AAV2-hCHM)	Spark Therapeutics Nightstar Therapeutics	An AAV2 vector transferring REP1 gene into the eye. The drug is delivered surgically by injection into the sub-retinal space	Retinal Gene Therapy for choroideremia	NCT02553135 NCT02077361 NCT03507686 NCT03496012 NCT01461213 NCT03584165
ADVM-043 (AAVrh.10hA1AT)	Adverum	An AAV expressing Alpha-1 Antitrypsin (A1AT) gene to patients with A1AT deficiency	A1AT Deficiency	NCT02168686 NCT03804021
LYS-SAF302	Lysogene, Regenxbio, Sarepta Therapeutics	AAVrh10 virus expressing N-sulfoglucosamine sulfohydrolase (SGSH)	Sanfilippo type A syndrome	NCT03612869 NCT02746341
AMT-061	uniQure	An AAV5 viral vector expressing the Padua variant of Factor IX (FIX-Padua)	Hemophilia B	NCT03569891 NCT03489291
Fidanacogene elaparvovec (SPK-9001)	Pfizer	An AAV with engineered capsid that expresses human coagulation factor IX gene	Hemophilia B	NCT03587116 NCT03307980
OTL-103 (GSK2696275)	Orchard Therapeutics	*Ex vivo* autologous transplantation of hematopoietic stem cells manipulated by a lentiviral vehicle expressing WAS gene	Wiskott Aldrich syndrome	NCT01515462 NCT03837483
OTL-200 (GSK2696274)	Orchard Therapeutics	*Ex vivo* autologous transplantation of CD34+ stem cells manipulated by lentiviral vector expressing human arylsulfatase A (ARSA) gene	Metachromatic Leukodystrophy (MLD)	NCT03392987
Instiladrin (rAd-IFN/Syn3)	FKD Therapies, Ferring Pharmaceuticals	Nonreplicating recombinant adenovirus type 5 expressing interferon alpha-2b (IFNα2b) gene, fused with the excipient Syn3 (rAD-IFN/Syn3)	Bacillus Calmette-Guérin (BCG)-unresponsive nonmuscle invasive bladder cancer (NMIBC)	NCT02773849
OTL-101	Orchard Therapeutics	Lentiviral transduced CD34+ cells to express ADA gene	Adenosine deaminase severe combined immune deficiency (ADA-SCID)	NCT03765632
RT-100 (Ad5.hAC6)	Renova Therapeutics	AAV5 vehicle expressing human adenylyl cyclase type 6 which it is directly injected throughout cardiac catheterization into the arteries feeding the heart	Heart failure and reduced ejection fraction	NCT03360448 NCT00787059
ADXS11-001 (Axalimogene filolisbac)	Advaxis	Targeted immunotherapy /attenuated Listeria monocytogenes manipulated for secretion of antigen/adjuvant fusion proteins	Metastatic cervical cancer	NCT02853604 NCT02002182 NCT02164461
OXB-301 (MVA-5T4)	Oxford BioMedica	An attenuated engineered vaccinia virus Ankara that delivers the 5T4 oncofetal antigen gene to stimulate the immune cells against cancer cells	Ovarian cancer, colorectal cancer	NCT01556841 NCT01569919
Pexa-Vec (JX-594)	SillaJen, Transgene	Wyeth strain vaccinia virus engineered to directly lyse tumor cells and stimulate anti-tumor immunity	Hepatocellular carcinoma	NCT02562755 NCT03071094
SPK-8011	Spark Therapeutics	Manipulated AAV vehicle (to specifically transfer the human factor VIII (hFVIII) gene into liver	Hemophilia A	NCT03003533 NCT03432520 NCT03876301
Toca 511 and Toca FC (vocimagene amiretrorepvec)	Tocagen, ApolloBio	Toca 511 is a retroviral vector expressing cytosine deaminase. Toca FC is prodrug 5-fluorocytosine which is converted into 5-fluorouracil by CD	Glioblastoma Multiforme And Anaplastic Astrocytoma	NCT02414165 NCT02576665 NCT02598011
VB-111 (ofranergene obadenovec)	Vascular Biogenics	Targeted anticancer gene-based biologic administered as an IV infusion once every 2 months. VB-111 was developed through VBL’s Vascular Targeting System (VTS™). VBL says the mechanism combines blockade of tumor vasculature with an anti-tumor immune response	Solid tumors, including recurrent platinum-resistant ovarian cancer	NCT03398655
VGX-3100	Inovio Pharmaceuticals	A synthetic DNA vehicle targeting the E6 and E7 proteins of HPV types 16 and 18, also is considered as a DNA vaccine	Cervical high-grade squamous intraepithelial lesion infected by HPV subtypes of 16 and 18	NCT03185013 NCT03721978 NCT03603808 NCT03499795 NCT03180684.
VM202	ViroMed	DNA vehicle expressing two isoforms of hepatocyte growth factor, HGF728 and HGF723	Painful diabetic peripheral neuropathy, Chronic nonhealing ischemic foot ulcer in diabetes, Critical limb ischemia, Amyotrophic lateral sclerosis, Acute myocardial infarction	NCT02563522 NCT02427464 NCT03363165 NCT03404024
QTA020V	Astellas	A rAAV transfects retinal ganglion cell to express BDNF and one of its receptors (TrkB) resulting reduction in cell death	Glaucoma	–
EB-101	Abeona Therapeutics	This drug was designed to transfer a healthy copy of COL7A1gene using the patients’ own skin cells	RDEB (skin disease)	NCT01263379
AT132	Audentes Therapeutics	An AAV8 vector containing a functional copy of the MTM1 gene	X-Linked Myotubular Myopathy (ASPIRO)	NCT03199469
NSR-RPGR	Biogen/Nightstar	An AAV8 encoding retinitis pigmentosa GTPase Regulator (RPGR)	X-linked retinitis pigmentosa (XLRP)	NCT03116113
Generx (Ad5FGF-4)	Angionetics, Huapont Life Sciences	A human serotype 5 adenovirus that express fibroblast growth factor-4 (FGF-4) gene	Angina, Stable	NCT02928094 NCT01550614 NCT00346437
GS010	GenSight Biologics	AAV2 vector that expresses the human wild-type ND4 protein	Leber Hereditary Optic Neuropathy (LHON) caused by mutation of the ND4 gene	NCT02652780 NCT02652767 NCT03293524 NCT03406104

In recent years a number of gene therapies based on gene editing tools, especially CRISPR system, advanced to human clinical trial stage. Gene editing technologies including CRISPR, Zinc Finger Nuclease (ZFN) and TALEN allow scientists to undertake precise genomic modifications at desired human genome positions yielding tremendous beneficial results in modern medicine and the field of genetics. Ongoing human gene therapy trials based on gene editing systems are listed in **Table 3**. The upcoming trials mark the maturation of the gene editing tools into a clinical-grade technology.

**Table 3:** Recruited clinical trials based on genome editing technologies (e.g. CRISPR/Cas, ZFN and TALEN).

Product name	Developer/ sponsor	Structure and mechanism of action	Therapeutic indication and target tissue	Clinical trials ID
AGN-151587 (EDIT-101)	Allergan	An AAV5 vector employed in EDIT-101 contains two gRNAs and Cas9 to correct IVS26 mutation in CEP290 gene	Leber Congenital Amaurosis 10 (LCA10)	NCT03872479
Cyclophosphamide (PD-1 Knockout T Cells)	Sichuan University	*Ex vivo* gene manipulation of T cells with CRISPR/Cas system to target non-small cell lung cancer	Metastatic NSCLC	NCT02793856
PD-1 Knockout T Cells	Yang Yang	*Ex vivo* gene manipulation of Peripheral blood lymphocytes with CRISPR/Cas system to knockout PD1 gene. Then EBV-CTL will be produced (PD-1 Knockout EBV-CTL)	Advanced-stage EBV-associated malignancies	NCT03044743
PD-1 Knockout T Cells	Hangzhou Cancer Hospital	*Ex vivo* gene manipulation of T cells with CRISPR/Cas system to target esophageal cancer	Esophageal Cancer	NCT03081715
CTX001	Vertex Pharmaceuticals Incorporated	Autologous CD34+ Hematopoietic Stem and Progenitor Cells (hHSPCs) manipulated with CRISPR/Cas9 system at the enhancer position of the BCL11A gene leads to increase in fetal hemoglobin	Hematologic diseases (Hemoglobinopathies)	NCT03655678 NCT03745287
SB-FIX	Sangamo Therapeutics	An AAV2/6 virus with ZFN inserting Factor 9 gene under the control of albumin promoter for liver expression	Hemophilia B	NCT02695160
ST-400	Sangamo Therapeutics	Autologous Hematopoietic Stem Cell Transplant/ST-400 is composed of the patient’s own blood stem cells which are genetically modified with ZFN technology to disrupt a precise and specific sequence of the enhancer of the BCL11A gene	Transfusion Dependent Beta-thalassemia	NCT03432364
CCR5 gene modification	Affiliated Hospital to Academy of Military Medical Sciences	CD34+ hematopoietic stem/progenitor cells from donor are manipulated with CRISPR/Cas9 aiming CCR5 gene deletion from cell surface	HIV-1-infection	NCT03164135
SB-318	Sangamo	An AAV2/6 viral vehicle combined with ZFN to incorporated IDUA under the control of albumin promoter for liver expression	MPS I (Hurler syndrome)	NCT02702115
SB-913	Sangamo	An AAV2/6 viral vehicle linked with ZFN tool to incorporated IDS gene under the control of albumin promoter in liver cells	MPS II (Hunter’s syndrome)	NCT03041324
UCART123	Cellectis S.A.	Allogeneic engineered T-cells with TALEN system expressing anti-CD123 chimeric antigen receptor	AML	NCT03190278
UCART019	Chinese PLA General Hospital	CRISPR/Cas9 mediated CAR-T Cells Targeting CD19 in individuals with relapsed or refractory CD19+ leukemia and lymphoma	leukemia and lymphoma	NCT03166878
Anti-Mesothelin CAR-T cells	Chinese PLA General Hospital	Knocking out of PD-1 and TCR genes using CRISPR/Cas in CAR T Cells	Solid Tumor, Adult	NCT03545815
NY-ESO-1 redirected autologous T cells with CRISPR edited endogenous TCR and PD-1	University of Pennsylvania	Autologous T cells manipulated with a lentiviral vector for expressing NY-ESO-1 and transfected with CRISPR guide RNA to for knocking out of endogenous TCRα, TCRβ and PD-1 (NYCE T Cells)	Multiple Myeloma	NCT03399448
Universal Dual Specificity CD19 and CD20 or CD22 CAR-T Cewlls	Chinese PLA General Hospital	Using CRISPR/Cas9 to targetCD19 and CD20 or CD22 in CAR-T Cells for treatment of Relapsed or Refractory Leukemia and Lymphoma	Leukemia Lymphoma	NCT03398967

Recent advances in understanding molecular mechanism of human diseases and treatment are boosting the global gene therapy market. This market is categorized into cancers, neurological diseases, rare genetic diseases, cardiovascular disorders, and infectious diseases. Cancers and monogenic diseases had the highest market share in recent years respectively. Viral vectors (mainly retrovirus, lentivirus, adeno-associated virus) in comparison to non-viral vectors are the preferred gene therapy vehicles in the clinic. High-efficiency of gene transduction, specific gene delivery and targeting, safety and reduced administration dose are the main benefits of viral vectors. North America and Europe are dominant players and drive advancements in gene therapy market of cancer and rare genetic diseases. Nowadays, the key trends of the gene therapy market are high prevalence of human cancers and genetic disorders, clarifying gene therapy guidelines and rising financial support of gene therapy and cell-based gene therapy in clinical trials. However, safety and efficacy problems, prolonged laboratory procedures for conducting clinical studies, unknown product interactions with host, and high cost of gene therapy drugs are major barriers in the way of gene therapy market.

## Conclusion

Despite considerable efforts in gene therapy segment, only a few of the twenty approved gene and cell-based gene therapy products were translated into the clinic (May 2019). In the previous year, numerous promising results attested progress in clinical gene therapies for monogenic diseases, inherited blindness, certain inherited neurodegenerative diseases, metabolic genetic disorders and a number of bone marrow and lymph nodes cancers (Dunbar et al., 2018).

Growth and development of viral delivery systems emerge as effective tools for gene manipulation and gene therapy approaches such as CRISPR/Cas has revolutionized the realm of gene therapy (Dicarlo et al., 2017; Ehrke-Schulz et al., 2017).

Although Glybera was withdrawn from the European market in early 2017, approval of six gene therapy and cell-based gene therapy products, known as Invossa, Luxturna, Kymriah, Yescarta, Patisiran, and recently approved Zolgensma (May 2019), promise a new era in gene therapy for untreatable genetic disorders.

As such, the global gene therapy market has grown commensurately in recent years and is expected to grow at a high rate through 2030 according to Grand View Research. A recent report published by Roots Analysis on ‘Gene Therapy Market (2nd Edition), 2018-2030’, stated that nearly 300 product candidates are currently under various stages of development for a diverse range of applications.

## Author Contributions

All authors listed have made substantial, direct, and intellectual contribution to the work and approved it for publication.

## Conflict of Interest Statement

The authors declare that the research was conducted in the absence of any commercial or financial relationships that could be construed as a potential conflict of interest.
